# CORESS Feedback: Cases from the Confidential Reporting System for Surgery

**DOI:** 10.1308/rcsann.2025.0029

**Published:** 2025-05-01

**Authors:** H Corbett

**Affiliations:** on behalf of the CORESS Advisory Board

## Abstract

CORESS is an independent charity. The online reporting form is available via the CORESS website (coress.org.uk), which also includes previous Feedback reports. Published cases are acknowledged by a Certificate of Contribution, which may be included in the contributor’s record of continuing professional development, or which may form part of appraisal or annual review of competence progression portfolio documentation. Contributions from surgeons in training are particularly welcome.

In a joint safety initiative, Getting It Right First Time (GIRFT), NHS England and the Royal College of Surgeons of England have produced best practice guidance for documentation of the following procedures: laparoscopic appendicectomy, laparoscopic cholecystectomy, inguinal hernia repair, laparotomy and laparoscopic bowel resection, and thyroidectomy. The following four cases are vignettes that were provided by GIRFT and that have been discussed by the CORESS advisory board. The events in these cases underpin the need for comprehensive and thorough documentation for these procedures.

## Injury from insertion of laparoscopic port

### Case 303

The patient had a complex surgical history, with multiple abdominal procedures including hysterectomy, fundoplication for reflux, cholecystectomy and incisional hernia repair. The patient developed an inguinal hernia and was admitted to hospital for laparoscopic repair. After the port was inserted into the patient’s abdomen, the blood pressure dropped, the procedure was converted to a laparotomy, a perforation of the left common iliac artery was identified and the artery was clamped. However, the patient suffered a hypoxic brain injury as a result of the bleeding from the artery, leaving them with significant disability, including mobility and cognitive problems.

An operation note was not completed by the surgeon (because the complication arose after insertion of the port and the surgery therefore never really got underway) and, in the absence of any alternative contemporaneous explanation, it had to be accepted that the arterial damage was probably caused by the surgeon inserting the port too far into the abdomen. Early admission of liability was made on that basis and substantial damages exceeding £3 million were paid to the claimant. Legal costs were in the region of £500,000.

#### GIRFT message

A detailed operative note is required regardless of at which point in the procedure a complication occurs. When inserting laparoscopic ports, key details including anatomical location, technique used (including whether under direct vision) and the pressure set should be recorded. Recording any intraoperative complications (including the findings and what action was taken to remedy them), noting conversion from a laparoscopic procedure to laparotomy, and recording any additional procedures performed (together with the rationale for them) is advised.

#### CORESS comments

A comprehensive operation note forms a fundamental component of communication in the continuity of surgical care beyond the operating theatre to all stations on the subsequent patient pathway. The Royal College of Surgeons of England has set out in detail the components of a good operation note in its *Good Surgical Practice* guidance.^1^ Further relevant details are provided by Hoggett *et al* in *How to write an operation note*.^2^ The CORESS advisory board noted that it was particularly important to document untoward, unusual or adverse occurrences, in the event that documentation is relied on subsequently for medicolegal purposes. Where feasible, a drawing or illustration may greatly enhance a reader’s understanding of the procedure undertaken.

References1.Royal College of Surgeons of England. Good Surgical Practice. https://www.rcseng.ac.uk/standards-and-research/gsp/
(cited March 2025).2.Hoggett
L, Wright
A, Wilson
J. How to write an operation note. *BMJ*
2017; **356**: j355.

## Incomplete removal of appendix

### Case 304

A patient underwent a laparoscopic appendicectomy, which was uncomplicated. He returned 18 months later with further right-sided pain. Having been investigated for over one year, he was admitted for a laparoscopic stump appendicectomy. At surgery, the patient was found to have an appendix approximately 12.5cm in length. This was explained as duplicate appendicitis; however, there was no evidence for this. The likely explanation was an incomplete appendicectomy but surgical records were scant. A clinical negligence case was brought and admissions had to be made in the absence of a clear documentation to the contrary.

#### GIRFT message

Documenting operative findings including any stump level left and operative technique, with detailed and clear description, is important, not just in potentially defending a later claim but more importantly in managing ongoing patient care when postoperative difficulties are suffered.

#### CORESS comments

CORESS noted that there appeared to be no match of pathological records to the procedure. It was also discussed that keeping a pictorial record at the time of the laparoscopic appendicectomy might have facilitated a defence in this case. Finding the appendix may be difficult at appendicectomy. Mobilisation of the caecum allows identification of the taeniae coli, the three separate bands of smooth muscle that converge on the vermiform appendix and may aid identification of this structure.

## Alleged nerve injury during laryngectomy

### Case 305

A patient underwent laryngectomy and left hemi-thyroidectomy owing to a laryngeal tumour. Although the operation was a success, the patient unfortunately suffered a hypoglossal nerve injury, which affected speech and ability to swallow. Detailed operative records showed no evidence of negligence and the claim, brought subsequently for clinical negligence, was defended successfully at trial.

#### GIRFT message

Sometimes events occur that are not expected, are rare and cannot necessarily be explained. Depending on the circumstances of the case, wholly unexpected events are not negligent. While this case was, rightly, defended with the benefit of impressive witness evidence, this would have not been possible without the detailed operative note that clearly identified key structures that had been appropriately protected. The opportunity was still taken among clinical staff to reflect on what might be done in future to avoid any adverse outcome by ensuring all documentation has sufficient detail to defend such a claim.

#### CORESS comments

CORESS agreed that some adverse incidents are recognised complications of surgical procedures. In addition to keeping scrupulous operative records that record identification and protection of structures at risk, comprehensive preoperative consent covering all potentially likely complications should be undertaken.

## Failure to interpret operative cholangiogram correctly

### Case 306

A patient was admitted for a laparoscopic cholecystectomy. A large stone had been identified in the gallbladder and this proved difficult to retract. Calot’s triangle was fully dissected and the presumed cystic duct was tied distally. On-table cholangiography was undertaken. The operative cholangiogram was interpreted wrongly, leading to excision of the bile duct, with transection in its distal portion and proximally, in the hilum of the liver. The above was not recognised during the operation and the patient was subsequently discharged. All appeared to have gone well. The patient then suffered severe pain and was readmitted with sepsis. The patient had to undergo further (and this time urgent) surgery, where the previous error was recognised and addressed. Admission to intensive care was then required.

Unfortunately, the negligent interpretation of the cholangiogram and therefore inaccurate documentation of the intraoperative findings did lead to considerable ongoing difficulties for this patient. While not appreciated at the time, it did not take long for clinicians to reflect and for an early admission of substandard care to follow. Liability was admitted at an early stage and the clinical negligence claim was swiftly resolved. An apology from the trust’s chief executive was provided.

#### GIRFT message

Recognition of an error by the surgeon, who was prepared to go on record to diagnose the complication and admit liability, allowed the patient to receive the right treatment for the complication and the claim to be resolved quickly, reducing the risk of additional costs for failure to follow up.

#### CORESS comments

If a cholangiogram is requested, it should be interpreted correctly. It was noted that this could have been a case of confirmation bias (i.e. if new information is provided that does not fit with an established preconception, then this new information may be disregarded). It was also noted that bile duct injuries of this nature should be dealt with by specialist centres and not by the surgeon who has caused the primary injury.

## Fournier’s gangrene fatality

### Case 312

A 59-year-old diabetic patient with end-stage renal failure underwent live unrelated donor kidney transplantation. Four months later, four days after developing an indurated perianal swelling, he was admitted to hospital with cellulitis of his scrotum spreading into the lower part of his anterior abdominal wall. Initially, he required insulin to control hyperglycaemia, and he was commenced on a broad-spectrum intravenous antibiotic and intravenous fluids. Surgery was delayed for 12 hours owing to theatre availability and during that period, he developed blistering and incipient skin was necrosis of the lower part of the abdominal wall. On palpation, crepitus was noted in the abdominal wall, extending above the cellulitic area. A cephalosporin and metronidazole, to cover Gram-negative anaerobes, were added to the antibiotic regime.

The patient was taken to theatre, where a perianal abscess was drained, and debridement of the affected skin of the abdominal wall and scrotum was undertaken, with specimens sent for microbiological cultures. A vacuum dressing was applied to the area of the skin defect. However, over the subsequent 24 hours, the cellulitis and skin necrosis continued to spread up the abdominal wall. A re-exploratory procedure with full-thickness resection of the abdominal wall was performed, with sterile packing of the ensuing laparostomy, but the patient deteriorated rapidly and succumbed the following day.

#### Reporter’s comments

•  There was delay in recognising the manifestations of Fournier’s gangrene. The condition requires a multimodal approach with haemodynamic stabilisation, broad-spectrum antibiotics and early surgical debridement.
•  Diabetes and immunosuppression are common aetiological factors for Fournier’s gangrene.
•  Delay in surgical intervention may have contributed to the rapid extension of the affected tissue and poor outcome.
•  The full extent of the disease may not be apparent from the area of cutaneous involvement, which is usually less than the subcutaneous disease.

#### CORESS comments

Fournier’s gangrene is a fulminant form of necrotising fasciitis commonly affecting perineal, perianal or genital regions but that may spread to the abdominal wall, moving along fascial planes. It is a mixed infection caused by both aerobic and anaerobic bacterial flora, and associated with rapid spread and frequently also with multisystem organ failure and death. A high index of suspicion with early diagnosis and treatment by haemodynamic stabilisation, surgical debridement and antibiotics is essential although mortality remains high. Immunosuppression, diabetes and alcohol misuse are recognised risk factors.

**Figure 1:**
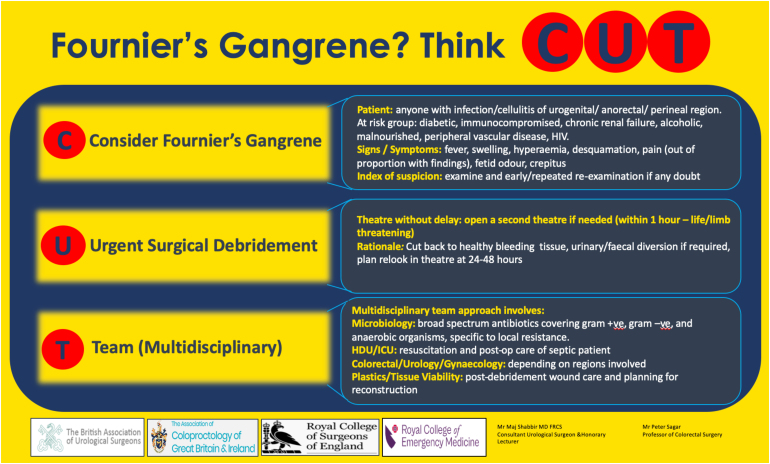
Alert sheet for Fournier’s gangrene with the mnemonic CUT

The Fournier’s gangrene severity index developed by Laor *et al* has been shown to predict mortality.^1^ Various royal colleges have jointly produced an alert sheet with the mnemonic CUT (Figure 1) to focus consideration on steps in the management of Fournier’s gangrene:

•  C – Consider Fournier’s gangrene (risk groups and high index of suspicion)
•  U – Urgent surgical debridement back to healthy bleeding tissue
•  T – Team (multidisciplinary approach)

Reference1.Laor
E, Palmer
LS, Tolia
BM
*et al*. Outcome prediction in patients with Fournier’s gangrene. *J Urol*
1995; **154**: 89–92.7776464


## Wrong site excision of skin lesion

### Case 315

A 56-year-old woman was referred by her general practitioner for specialist opinion after discovering an abnormal skin lesion on her shoulder. A basal cell carcinoma was suspected.

The patient was seen in the outpatient clinic at her local hospital and referred for surgery. A series of administrative errors and miscommunications delayed her operation date, which was finally scheduled three months after her initial referral.

When the patient attended for surgery, she was told that the consultant surgeon, whom she had seen in the outpatient department and whom she had expected to perform the operation, was on holiday. Another staff-grade surgeon would perform the procedure. The surgeon who was to perform the operation had been expecting a day in clinic. On arrival at work, she discovered she would be operating instead. She was running late. A new pre-list briefing session had been introduced during the previous week following a Care Quality Commission inspection. This further delayed the list start time as people were unfamiliar with the new process. The operation site was not marked before the patient went into the operating theatre. Local anaesthesia was administered and the operation was performed. It was later discovered that the checklist recorded the site as “marked”, the form having been completed ahead of the list to ‘save time’.

Histopathology reported that the lesion was non-malignant. A second procedure, wide local excision, was therefore not considered necessary.

Three days following the procedure, the wound dressing came loose. The patient examined the wound area, which was very red. She noticed that the suture line was in a position away from the original lesion area and realised that the original lesion of concern was still present.

The patient arranged an urgent follow-up appointment and was seen by the operating surgeon, who, although acknowledging that she had operated on a lesion that the patient had not expected, did not admit that a mistake had been made. A further biopsy was arranged for the following week, at which the original lesion was excised. The original lesion proved positive for basal cell carcinoma.

#### CORESS comments

This case represents a ‘never event’. There were clear deficiencies in adhering to procedural checks as outlined in the 2023 National Safety Standards for Invasive Procedures from the Centre for Perioperative Care^1^ and in the British Society for Dermatological Surgery/British Association of Dermatologists surgical checklist guidelines.^2^ In addition, the clinician did not adhere to duty of candour guidance with the patient. The Royal College of Surgeons of England has a useful free e-learning module on the duty of candour, which can be accessed via:

www.rcseng.ac.uk/standards-and-research/standards-and-guidance/good-practice-guides/duty-of-candour

References1.
Centre for Perioperative Care. The National Safety Standards for Invasive Procedures (NatSSIPs). https://cpoc.org.uk/guidelines-resources-guidelines/national-safety-standards-invasive-procedures-natssips
(cited March 2025).2.
British Society for Dermatological Surgery. BSDS/BAD guidelines. https://bsds.org.uk/resources/bsds-bad-guidelines
(cited March 2025).The following case was taken from the 2014 NHS England Never Events Taskforce report *Standardise, Educate, Harmonise: Commissioning the Conditions for Safer Surgery*.

## Checklist problems 1

### Case 316

A patient had a bradycardic cardiac arrest on the ward four days after an aortic valve replacement. There was a considerable delay in restoration of cardiac output as the leads for a pacemaker box and the leads for external pacing/defibrillation were absent from the resuscitation trolley. The trolley and its contents are safety checked each morning against a checklist where each item is ticked as present. The leads were ticked as present but were not in fact there, and they had not been removed in the period between the check and the cardiac arrest either.

#### Reporter’s comments

This highlights potential issues with often repeated checklists where checklist fatigue can set in and complacency may occur, especially when items on the trolley are usually present. Checklists work well up until the point where they fail. Feedback obtained was that the checklist was time-consuming and complex to complete. Staff felt that the checks were unnecessary as usually, nothing was wrong. Checklists reduce the risk of adverse incidents although they do not eradicate risk altogether. However, failure to use a checklist or incorrect usage increases the risk of harm.

#### CORESS comments

CORESS agreed with the reporter’s comments on checklist fatigue and the risk of paying lip service to a list without actually undertaking the required checks. This incident is a valuable reminder of the purpose of a checklist and also of the potential for lack of engagement if staff cannot perceive that purpose. Involvement of staff in design of the checklist has been shown to improve checklist implementation.

## Checklist problems 2

### Case 317

A four-year-old child was listed for umbilical hernia repair. On the day of surgery, she was upset and difficult to examine. In an attempt to placate her, the surgeon in training drew a midline ‘smiley face’ as a site mark in the epigastrium. Consent was taken for umbilical hernia repair.

The child came to theatre with her parent and was checked in although the site mark was not inspected. Once in theatre, the ‘time out’ was performed before the surgeon scrubbed in, and the patient was prepared and draped. The surgeon in training scrubbed in with a junior colleague and started the procedure, making an incision in the epigastrium at the site of the site mark. The consultant entered theatre just after the incision was made and noted the error. The consultant then scrubbed in and undertook the correct procedure. The family was informed and received feedback from the root cause analysis investigation.

#### Reporter’s comments

Two things stood out when the case review was undertaken. First was the site mark, which was distracting. The surgeon in training reflected that they thought that the child was having an epigastric hernia repair as they commenced the incision. The second factor was that despite undertaking appropriate checklists, this failed to prevent what was classified as a never event (wrong site surgery).

#### CORESS comments

Surgical standards for incision site marking are outlined in detail in the 2023 National Safety Standards for Invasive Procedures from the Centre for Perioperative Care.^1^ Site marking is covered in the preoperative World Health Organization surgical safety checklist and this was an opportunity to have clarified that the proposed incision corresponded with the operation to be undertaken.

Reference1.
Centre for Perioperative Care. The National Safety Standards for Invasive Procedures (NatSSIPs). https://cpoc.org.uk/guidelines-resources-guidelines/national-safety-standards-invasive-procedures-natssips
(cited March 2025).
